# Emerging roles of plasmacytoid dendritic cell crosstalk in tumor immunity

**DOI:** 10.20892/j.issn.2095-3941.2023.0241

**Published:** 2023-10-09

**Authors:** Leilei Yang, Songya Li, Liuhui Chen, Yi Zhang

**Affiliations:** 1 Biotherapy Center, The First Affiliated Hospital of Zhengzhou University, Zhengzhou 450052, China; 2 Department of Stomatology, The First Affiliated Hospital of Zhengzhou University, Zhengzhou 450052, China

**Keywords:** Plasmacytoid dendritic cell, tumor microenvironment, cell crosstalk, immune activation, immune suppression

## Abstract

Plasmacytoid dendritic cells (pDCs) are a pioneer cell type that produces type I interferon (IFN-I) and promotes antiviral immune responses. However, they are tolerogenic and, when recruited to the tumor microenvironment (TME), play complex roles that have long been a research focus. The interactions between pDCs and other components of the TME, whether direct or indirect, can either promote or hinder tumor development; consequently, pDCs are an intriguing target for therapeutic intervention. This review provides a comprehensive overview of pDC crosstalk in the TME, including crosstalk with various cell types, biochemical factors, and microorganisms. An in-depth understanding of pDC crosstalk in TME should facilitate the development of novel pDC-based therapeutic methods.

## Introduction

Immune evasion is a distinguishing characteristic of solid tumors^1^. Tumor cells use various mechanisms, such as the expression of immune checkpoint molecules and recruitment of immunosuppressive cells, to circumvent the immune system, thereby promoting their own growth^2–5^. Immune checkpoint blockade (ICB) therapy, recently designed for blocking tumor immune escape, activates the anti-tumor immune response and has been approved for treating multiple tumor types, thus shifting the traditional paradigm of tumor therapy^6^. For some patients with tumors, ICB treatment can have lasting clinical efficacy. However, the overall response rate of ICB treatment is low^7,8^. Currently, approximately 80% of patients show a lack of response, or develop adaptive resistance, to ICB treatment^7,8^, because tumor cells, in addition to expressing immune checkpoint molecules, can achieve immune escape through various complex mechanisms^3,4,9^. Tumor cells can recruit and induce immune active cells into immunosuppressive cells, thus building an immunosuppressive microenvironment facilitating immune escape^4,10^. Therefore, analyzing the crosstalk between immunosuppressive cells and the tumor microenvironment (TME), and further exploring their specific mechanisms in regulating anti-tumor immunity, may provide new targets and therapeutic strategies for anti-tumor immunotherapy.

**Figure 1:**
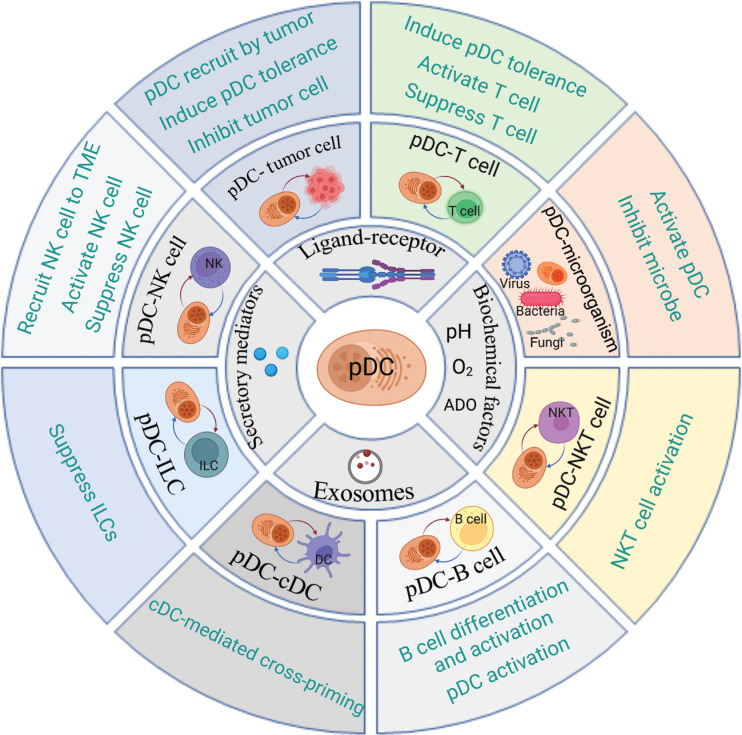
Schematic of plasmacytoid dendritic cell (pDC) crosstalk with other components in the tumor microenvironment (TME). In the TME, pDC crosstalk with other components through multiple patterns (the second ring) includes ligand–receptor conjunction, biochemical factors, exosomes, and secretory mediators. The pDC crosstalk targets (the third ring) in the TME include tumor cells, T cells, natural killer (NK) cells, innate lymphoid cells (ILCs), conventional dendritic cells (cDCs), B cells, NKT cells, and microorganisms. The subsequent functions (the fourth ring) of pDC crosstalk with these components are summarized. The figure was created with BioRender (BioRender.com).

Recent studies have revealed the presence of plasmacytoid dendritic cells (pDCs) within tumors, and highlighted their crucial roles in immune regulation and subsequent effects on anti-tumor immunity, which are garnering increasing attention^11–13^. pDCs are a subset of dendritic cells (DCs)^14^. During viral infection, activated pDCs secrete IFN-I, which exerts immune-stimulating functions, including inducing myeloid dendritic cell (mDC) maturation, activating natural killer (NK) cells, promoting antibody production by plasma cells, initiating type 1 T helper (Th1) cell proliferation, and inhibiting regulatory T cell (Treg) function and consequently priming antiviral immunity^14–16^. Moreover, pDC-derived IFN-I is involved in autoimmune disease pathogenesis^17^. However, several studies have shown that pDCs are highly plastic^18^. In the TME, pDCs undergo phenotypic and functional alterations that severely impair their IFN-I secretion and exert immunosuppressive effects through multiple mechanisms^14^. Tumor-infiltrating pDCs directly or indirectly interact with various cell types in the TME, thus providing the basis for their immunosuppressive function^17,19,20^. Therefore, comprehensive analysis of the regulation of the tumor-infiltrating pDCs to modulate the immune response through crosstalk with other components (summarized in **Figure 1**) has provided new insights and accelerated the translation of current knowledge into clinical practice.

## pDC overview

In 1958, pDCs were first observed in human lymph nodes, which possess plasma cell morphology and were originally denoted “T-associated plasma cells”^21^. Approximately 40 years later, pDCs were defined as a unique cell type that produces substantial amounts of IFN-I and may differentiate into conventional dendritic cells (cDCs)^22^. Over the next 2 decades, studies examined pDC development and biological roles^14,15,17^. The bone marrow continuously produces pDCs, which enter the blood with a non-activated phenotype^14^. During pDC development, the cytokine receptor fms-like tyrosine kinase 3 and its downstream transcription factor E2-2 play crucial roles in mediating the differentiation of pDCs from progenitors, and maintaining the pDC phenotype^23^. Disruption of E2-2 expression in pDCs leads to their spontaneous transformation into DCs^24^. After pDC generation and release into the peripheral blood, pDCs are recruited into lymph nodes and tissues^25,26^, where they play important roles in biological and pathological conditions such as viral infections and tumors^17,26^.

pDCs are a heterogeneous cell population^27–29^, and multiple pDC subtypes with diverse functions and distinct markers have been defined. For example, pDCs have been classified into 2 subtypes according to CD2 expression^27^: compared with CD2^low^ pDCs, CD2^high^ pDCs are more potent in priming T cells by secreting more IL12p40 and expressing higher levels of the co-stimulatory molecule CD80^27^. CD5 and CD81 have been used to further classify CD2^high^ pDCs^28^. Unlike pDCs, which are well known to secrete IFN, the CD2^high^CD5^+^CD81^+^ subpopulation produces almost no IFN-I after stimulation^28^. However, the CD2^high^CD5^+^CD81^+^ subpopulation strongly induces T cell proliferation, triggers B cell activation, and promotes Treg formation^28^. Moreover, pDCs activated after a single stimulus have been categorized into programmed cell death ligand 1-positive (PD-L1^+^) CD80^–^, PD-L1^+^CD80^+^, and PD-L1^+^CD80^–^ subtypes^29^. The PD-L1^+^CD80^–^ subtype has a plasmacytoid morphology and specializes in IFN-I secretion. The PD-L1^+^CD80^–^ subtype exhibits a dendritic morphology and adaptive immune function. The PD-L1^+^CD80^+^ subtype has both innate and adaptive functions. pDCs develop from both cDC progenitors and common lymphoid progenitors^14,30^. Using single-cell analysis, a recent study has shown that pDC origin determines the cells’ transcriptional and functional heterogeneity^31^.

pDCs are highly plastic. Activated pDCs trigger both innate and adaptive immune responses. High levels of peripheral circulating pDCs indicate better overall survival in patients with tumors^32,33^. An OX40^+^ pDC subtype, which has an immunostimulatory phenotype and exerts anti-tumor immune responses, has been found to be enriched in the TME^34^. However, numerous studies have shown that pDCs can transform into an immunosuppressive phenotype in tumors and facilitate the formation of a suppressive TME by expressing immune checkpoints and inducing Treg formation^18–20,35^. Studies have indicated that high pDC infiltration in the TME indicates poor prognosis in head and neck cancer, breast cancer, and ovarian cancer^11,12,36^. In addition, depletion of pDCs alleviates the immunosuppressive status of the TME and inhibits tumor progression^11,37,38^. Thus, several studies have focused on identifying how the TME educates pDCs and renders them immunosuppressive. However, the specific mechanism through which pDCs suppress tumor-infiltrating T cells and Tregs remains to be elucidated through analysis of the specific crosstalk of pDCs with other components in the TME. This investigations could markedly advance understanding of the TME and developing new pDC-based strategies for tumor immunotherapy.

## pDC–tumor cell crosstalk

**Figure 2:**
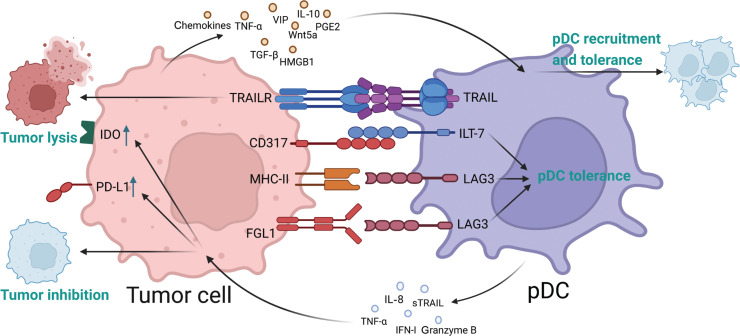
Crosstalk pattern between plasmacytoid dendritic cells (pDCs) and tumor cells. Tumor cells and pDCs affect each other through ligand–receptor ligation and mediator secretion. The figure was created with BioRender (BioRender.com).

During tumor progression, multiple pDCs are recruited and infiltrate the TME^13^. pDCs exhibit both tumor-promoting and tumor-inhibiting effects^13^. The specific mechanisms of pDC–tumor cell crosstalk might contribute to this divergent effect. In this section, the crosstalk between pDCs and tumor cells, including the effects of tumor cells on pDCs and vice versa, is comprehensively described (**Figure 2**).

### Effects of tumor cells on pDCs

#### Cell–cell contact

Cell–cell contact based on ligand–receptor interactions is a prominent pattern of intercellular communication^39,40^. In the TME, tumor cells exert direct effects on pDCs through ligand–receptor interactions. Notably, CD317 on tumor cells interacts directly with immunoglobulin-like transcript 7 (ILT-7) on pDCs^41^. CD317 (also known as BST-2, HM1.24, or tetherin), a representative IFN-I-induced protein that is highly expressed in multiple tumor types^42,43^, inhibits IFN-I secretion from pDCs and alters the phenotype of pDCs by interacting with ILT-7 on pDCs^41^. After this interaction, ILT-7 induces a calcium-dependent signaling cascade and inhibits the release of IFN-I and other proinflammatory cytokines from pDCs through its intracellular downstream immunoreceptor tyrosine-based activation motif^44^. Therefore, the CD317–ILT-7 signaling axis might act as a negative feedback regulatory loop preventing uncontrolled inflammatory responses; this loop could potentially be exploited by cancer cells to suppress the anti-tumor immune response. Importantly, somatic mutational analysis of tumor tissues has revealed that specific genetic changes in CD317 further enhance ILT-7-mediated IFN-I inhibition to the point of complete blockade of IFN-I production^41^. Therefore, CD317 mutations might plausibly have potent inhibitory functions within tumors. Additionally, our findings have demonstrated that a positive correlation between CD317 expression and the immunosuppressive state of the TME leads to unfavorable prognosis in head and neck squamous cell carcinoma (HNSCC)^42^. Thus, blocking the CD317–ILT-7 interaction might potentially increase IFN-I secretion and activate the anti-tumor immune response. This mechanism might enable the development of novel therapeutic methods for tumor immunotherapy.

Lymphocyte activation gene 3 (LAG3, CD223), the third clinically approved immune checkpoint target after cytotoxic T lymphocyte-associated antigen-4 (CTLA-4) and programmed cell death 1 (PD-1), is an immunosuppressive receptor expressed on the surfaces of human T and NK cells^45^. LAG3-mediated signaling impairs the anti-tumor immune response of human T and NK cells in the TME^46^. LAG3 expression on pDCs is 10-fold higher than that on activated T cells and Tregs^47^. Thus, LAG3 may have a more important function in regulating pDCs, in contrast to the well-established role of LAG3 in T and NK cells^47^. Major histocompatibility complex class II (MHC-II), which is expressed on antigen-presenting cells and a subset of tumor cells^48^, is the canonical ligand of LAG3^49,50^. The interaction between LAG3 on pDCs and MHC-II on tumor cells impairs IFN-α secretion and enhances IL-6 production, thus resulting in the formation of an immunosuppressive TME^51^. Moreover, multiple studies have shown that fibrinogen-like protein 1 (FGL1), another newly discovered ligand of LAG3 independent of MHC-II^52^, is upregulated in lung cancer, prostate cancer, melanoma, colorectal cancer, and breast cancer^52^. As a high-affinity LAG3 ligand, FGL1 binds LAG3 and consequently participates in a new immune checkpoint pathway inhibiting the T cell immune response^52^. However, the effects of LAG3–FGL1 interaction on the biological function of pDCs must be further investigated.

Many other important “checkpoint” molecules, including LAIR1, CD303, T cell immunoglobulin and mucin domain 3 (TIM3), NKp44, ITL7, and CLEC4A, are also expressed on human pDCs^53–56^. These underlying pDC–tumor cell interactions should be further confirmed to develop a complete picture of direct pDC–tumor cell crosstalk.

#### Crosstalk through secretory mediators

The tumor cell secretome encompasses growth factors, cytokines, enzymes, hormones, glycoproteins, coagulation factors, and extracellular vesicles, which, through interactions with other components, are important in the formation of an immunosuppressive TME^57^. Multiple studies have demonstrated that the tumor cell secretome also plays crucial roles in the crosstalk between pDCs and tumor cells, as comprehensively reviewed below.

Tumor cells produce multiple chemokines, including large amounts of CXCL12, which recruits pDCs by binding CXCR4 on pDCs in oral squamous cell carcinoma^58,59^. A previous study has indicated that circulating pDCs in patients with melanoma substantially express CCR6 and migrate to tumor sites by binding tumor cell-derived CCL20^60^. Moreover, pDCs express ChemR23 and are recruited in cervical metaplasia and dysplastic carcinoma sequences through ChemR23/chemerin ligation^61,62^. In addition, tumor and stromal tumor-associated cells release cytokines (such as CXCL10 and CXCL12) and chemokines (such as CCL2), which promote the migration of pDCs from the circulation to damaged tissue^63^. Together, these mechanisms may induce high pDC infiltration into the TME.

After recruitment to the TME, pDCs are further educated by the tumor cell secretome. High-mobility group box 1 (HMGB1) is a damage-associated molecular pattern that triggers immune responses during tissue damage and infection^64^. HMGB1 also induces immunosuppression and tumor progression, in which tumor cells secrete HMGB1, and Treg and monocyte cell suppression is promoted^65,66^. HMGB1 binds the receptor of advanced glycation end products (RAGE) on pDCs and alters their phenotype by decreasing the expression of mature pDC markers such as CD83, CD40, CD86, HLA-DR, CCR7, and CD11c^61^. HMGB1 also inhibits pDC maturation, thus decreasing IFN-α secretion after Toll-like receptor (TLR) 9 stimulation, and inducing a tolerogenic phenotype of pDCs^61^. The effects of HMGB1 on pDCs could potentially be reversed by treatment with anti-HMGB1 inhibitors or a blocking antibody targeting RAGE, thus suggesting a possible therapeutic perspective^61^. In addition to binding RAGE, tumor-secreted HMGB1 interacts with TIM3, a type I transmembrane protein, and consequently inhibits the transport of nucleic acids to endosomal vesicles, and weakens the anti-tumor effects of DNA vaccines and cytotoxic chemotherapy through the anti-nucleic acid-sensing system^67,68^. TIM3 is a co-inhibitory receptor expressed on T cells, Tregs, and innate immune cells (macrophages and dendritic cells)^68^. Notably, TIM3 is highly expressed in some pDCs^67^. Therefore, although no direct evidence has been reported, HMGB1 might possibly affect pDCs *via* TIM3.

Transforming growth factor β (TGF-β) is a pleiotropic cytokine with crucial roles in pathological and physiological conditions, including cancers^69,70^. Moreover, tumor cell-secreted TGF-β inhibits IFN-I production from pDCs^18^. One plausible mechanism underlying this inhibition involves the maintenance of lysosomal associated membrane protein 5 expression by TGF-β exposure, which promotes TLR9’s translocation into late endosomes and subsequent degradation, thereby limiting IFN-I secretion from pDCs^71,72^. TGF-β inhibition also rescues CD69, MHC-I, and pDC-TREM expression in CpG-activated pDCs^18^. Moreover, TGF-β affects pDCs by acting synergistically with other secretory mediators, including prostaglandin E2 (PGE2), TNF-α, and IL-10. PGE2 is an inflammatory mediator found in numerous cell types^73^, which increases tumor growth and invasion, decreases apoptosis, facilitates metastasis and angiogenesis, and inhibits anti-tumor immunity^74^. Tumor-derived PGE2 and TGF-β synergistically inhibit IFN-α and TNF-α production in stimulated pDCs. Furthermore, PGE2- and TGF-β-treated pDCs exhibit a “tolerogenic” phenotype with CD40 downregulation and CD86 upregulation. PGE2 and TGF-β also decrease the migration of TLR-stimulated pDCs into tumor-draining lymph nodes through downregulating the CCR7/CXCR4 ratio^75^. TNF-α belongs to the TNF/TNFR cytokine superfamily, which is commonly detected in biopsies of human cancers (such as epithelial tumors, ovarian cancer, and renal cancer). Moreover, tumor-derived TGF-β and TNF-α synergistically inhibit IFN-I and TNF-α production through blocking interferon regulatory factor 7 (IRF7) expression and nuclear translocation^76,77^. As an important immunosuppressive factor in the TME^78^, IL-10 acts synergistically with TGF-β, thus decreasing TLR9 mRNA expression in human peripheral blood pDCs and inhibiting TLR9-mediated IFN-α generation by pDCs^79^. Moreover, IL-10 enhances the suppressive effects of tumor cell supernatants on IFN-α secretion from pDCs^78^.

Cancer cells secrete non-canonical wingless-related integration site 5a (Wnt5a), a homolog of the wingless protein in *Drosophila* species, which has tumor-promoting effects in melanoma, pancreatic cancer, and non-small cell lung cancer^72,80,81^. This protein also inhibits CD80 and CD86 upregulation in pDCs and IFN-I secretion by stimulating pDCs, possibly through preventing the cytoskeletal rearrangement required for pDC activation^80^.

Vasoactive intestinal peptide (VIP) is a neuropeptide secreted by a specific type of neuroendocrine tumor^72,82^. The VIP receptors VPAC1 and VPAC2 are expressed in human pDCs^83^. VIP inhibits IFN-α secretion and MHC-I expression in pDCs. However, VIP enhances the expression of activation markers, including CD86, MHC-II, and CCR7, in pDCs^83^. Moreover, VIP has been found to enable pDCs to trigger a T cell-based immune response toward Th2 *in vitro*^83^. We speculate that tumor-secreted VIP might affect tumor progression by interacting with tumor-infiltrating pDCs, a possibility warranting further investigation.

### Effects of pDCs on tumor cells

pDCs exhibit cytotoxic properties through TNF-associated apoptosis-inducing ligand (TRAIL)^84^. pDCs have been found to effectively lyse WM793 and SKMEL2 melanoma cells in a TRAIL-dependent manner, and the degree of lysis is associated with the expression of TRAIL receptors in melanoma cells^84^. In addition, pDCs directly induce tumor cell lysis *via* secreting cytotoxic cytokines including granzyme B (GZMB), TNF-α, and soluble TRAIL^85^. The function of GZMB in pDCs remains unclear. Some researchers have reported that GZMB is not involved in pDC-induced tumor cell killing^86,87^. Unstimulated GZMB^+^ pDCs do not lyse tumor cells^84^. Other cleavage molecules (such as perforin and granzyme) are required for GZMB-mediated effector functions^88^. However, pDCs express almost no perforins, granzymin, FasL, and lysozyme^84^. In contrast, GZMB^+^-activated pDCs effectively kill tumor cells^87^. These paradoxical findings may be due to methodological differences among studies^87^.

pDC-secreted IFN-I acts directly on tumor cells. IFN-I directly inhibits tumor cell proliferation and migration, and contributes to senescence and apoptosis^89^. In melanoma and breast cancer cells, IFN-I-driven TRAIL expression contributes to caspase 8-dependent apoptotic sensitivity to IFN-I^90,91^. In cervical cancer, IFN-I causes non-apoptotic proliferation arrest and early cytoplasmic accumulation of the anti-apoptotic proteins cFLIP and caspase 8^92^. The composition of the death-inducing signal complex activates caspase 8, thus leading to apoptosis^92^. In contrast, IFN-I causes immune suppression by promoting indoleamine 2,3-dioxygenase (IDO) and PD-L1 expression.^89,93,94^

### pDC-tumor cell crosstalk across different tumor types

Among tumor types, the etiology, immunogenicity, and immune-associated microenvironment are highly heterogeneous^95^, thereby resulting in profound differences in the fine-tuned pDC-tumor cell crosstalk. Human papillomavirus (HPV) infection is an etiological factor in some cancer cases^96,97^, and the TME in HPV positive tumors is distinct from that in HPV negative tumors^98–100^. A recent study has shown significantly dampened immune activating ability of pDCs in the HPV negative TME but not the HPV positive TME^101^. Moreover, virus-like particles have been found to activate tumor-infiltrating pDCs^102^. Thus, although direct evidence is lacking, pDC-tumor cell crosstalk may vary between HPV positive and negative tumors. In addition, the decrease in IFN-secreting ability of pDCs is considered a major indicator of pDC tolerance in the TME. Across tumor types, various tumor-derived factors have been identified to induce pDC tolerance (**Table 1**). For example, in HNSCC, tumor cells decrease the production of IFN-α by pDCs through the binding of CD317 and ILT-7, as well as the production of TGF-β, PGE2, and IL-10. In cervical cancer, tumor derived HMGB1 dampens the IFN-secreting ability of pDCs. Little research has directly investigated the variations in pDC-tumor cell crosstalk across diverse tumor types; therefore further studies are needed to understand pDC biology.

**Table 1:** pDC-tumor cell crosstalk across tumor types

Tumor type	pDC-tumor cell crosstalk	Reference
Head and neck cancer	Decrease in IFN-α production *via* CD317-ILT-7 and tumor-derived TGF-β, PGE2, and IL-10	^41,75,78^
Melanoma	pDC recruitment into the tumor microenvironment *via* tumor-derived SDF-1 and CCL20	^60,103^
	Decrease in IFN-α production *via* MHCII-LAG3, and tumor-derived TGF-β, PGE2, IL-10, and Wnt5a	^51,80,104^
	Tumor cell destruction *via* TRAIL-TRAILR, and pDC-derived granzyme B, TNF-α, and soluble TRAIL	^84^
	Induction of cell apoptosis *via* pDC-derived IFN-I	
Breast cancer	Decrease in IFN-α production *via* tumor-derived TNF-α and TGF-β	^77^
	Induction of cell apoptosis *via* pDC-derived IFN-I	^90^
Ovarian cancer	Decrease in IFN-α production *via* tumor-derived TNF-α and TGF-β	^105^
	Promotion of neoangiogenesis *via* pDC-derived TNF-α and IL-8	^106^
Cervical cancer	Decrease in IFN-α production *via* tumor-derived HMGB1	^61^
Lung cancer	Promotion of neoangiogenesis *via* pDC-derived IL-1a	^107^

## pDC–T cell crosstalk

Similarly to cDCs, mature pDCs present antigens to T cells and participate in T cell activation by expressing antigen-presenting and co-stimulating molecules, including MHC-II, CD40, CD80, and CD86^17,34^. In addition, pDC-secreted IFN-I stimulates T cells. In cancer, pDC-secreted IFN-I enhances anti-tumor CD8^+^ T cell effector function by increasing tumor-killing ability^108^. IFN-I also indirectly enhances anti-tumor CD8^+^ T cell responses by promoting the cross-presentation function of DCs^109^. However, the expression of IFN-I receptors on CD8^+^ T cells is downregulated in the TME, thereby inhibiting IFN-I-induced anti-tumor effects^110^.

**Figure 3:**
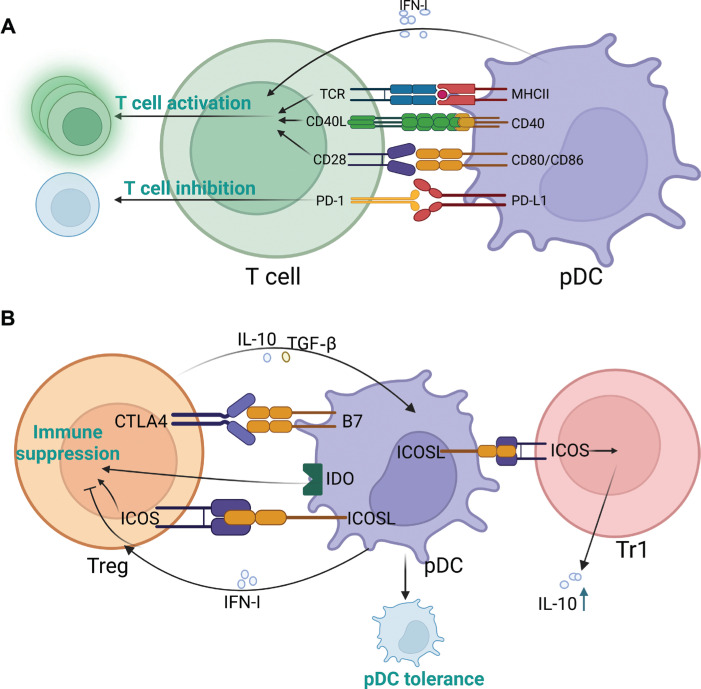
Crosstalk pattern of plasmacytoid dendritic cells (pDCs) and different types of T cells. (A) pDCs activate or inhibit T cells in multiple ways. (B) pDC–regulatory T cell (Treg) and pDC–type 1 regulatory T cell (Tr1) crosstalk patterns. pDCs exacerbate the immunosuppressive function of Tregs and Tr1s. The figure was created with BioRender (BioRender.com).

pDCs also inhibit T cell activation. When recruited to the TME, pDCs substantially express PDL1, which binds PD-1 on T cells and suppresses the T cell-mediated immune response^111^. However, owing to the lack of comparative experiments between PDL-1^+^ and PDL-1^−^ pDCs on T cells^111^, further exploration is needed. A schematic diagram of pDC crosstalk with CD4^+^ and CD8^+^ T cells is shown in **Figure 3A**.

Tregs, characterized by the expression of Foxp3, CD25, and CD24, are a T cell subset with substantial immunosuppressive effects. Tumor-infiltrating pDCs markedly express ICOS-L, which selectively binds ICOS on Tregs and promotes their Treg immunosuppressive function^112^. In tumor-draining lymph nodes, some pDCs express the tryptophan-degrading enzyme IDO, which primes undifferentiated CD4^+^ T cells to differentiate into Tregs *in vitro*^113^. Furthermore, IDO^+^ pDCs directly activate resting Tregs and stimulate their potent suppressive function^113^. B7 on pDCs interacts with CTLA4 on Tregs, thus enhancing IDO enzymatic activity. IDO-activated Tregs significantly promote PD-L1 and PD-L2 expression in DCs, and consequently suppress target T cells^113^. In addition, IFN-I produced by pDCs inhibits Tregs^114^. The inhibitory function of Tregs is inactivated by IFN-I through downregulation of cAMP levels in Tregs, thereby increasing the activation of effector T cells and the cytotoxicity of NK cells^114^. However, multiple studies have reported that the IFN-I secretion ability of tumor-infiltrating pDCs is largely diminished^13,18^. Tregs also affect tumor-infiltrating pDCs and secrete the suppressive cytokines IL-10 and TGF-β, which alter the pDC phenotype, inhibit immune activation through the classical pathway, impair IFN-α production by pDCs, and enhance tumor immunosuppression^79^. Thus, the effect of positive feedback between pDCs and Tregs enhances the immunosuppressive status of the TME.

In addition to Tregs, tumor-infiltrating pDCs exposed to tumor-derived factors cause naïve CD4^+^ T cells to differentiate into type 1 Tregs (Tr1)^115^. Tr1 cells, which were initially identified in individuals with long-term tolerance after allogeneic transplantation, are CD4^+^FoxP3^−^CD49b^+^LAG3^+^ cells that produce high levels of IL-10 and induce immunosuppression. Beyond pDC-induced Tr1 production, ICOS-L^+^ pDCs further promote IL-10 production by Tr1, thus driving the immunosuppressive status of the TME^115^. Blocking ICOS–ICOS-L ligation inhibits IL-10 production by Tr1 cells but has little effect on the immunosuppressive phenotype of Tr1^115^. A schematic diagram of pDC crosstalk with Tregs and Tr1 cells is shown in **Figure 3B**.

## pDC–B cell crosstalk

**Figure 4:**
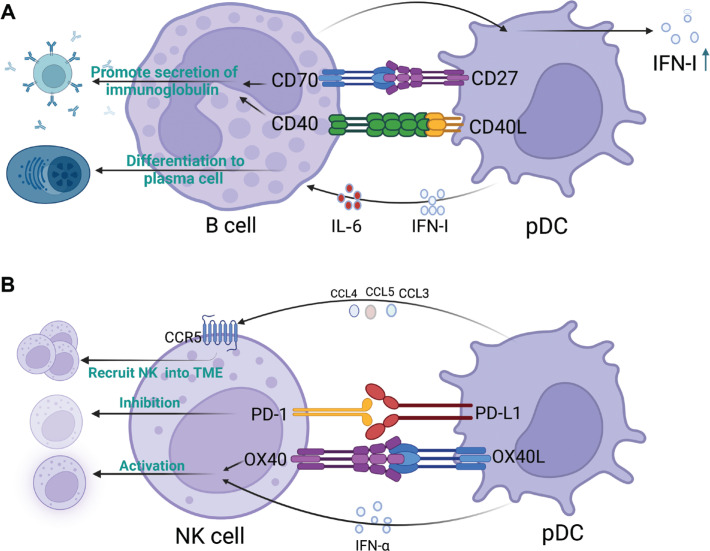
Crosstalk patterns of plasmacytoid dendritic cells (pDCs) with B cells and NK cells. (A) pDCs induce the differentiation and immunoglobulin secretion of B cells. B cells in turn promote pDC activation and IFN-I secretion. (B) Crosstalk pattern of pDCs and natural killer (NK) cells. pDCs recruit NK cells into the tumor microenvironment (TME), and both activate and inhibit NK cells. The figure was created with BioRender (BioRender.com).

In addition to the cells with clear crosstalk with pDC, other cells in the TME may interact with pDC; however, no experiments have definitively demonstrated such interactions within the TME. An antitumor immune response relies heavily on B cells, which are key effector cells in humoral immunity^116–118^. These cells inhibit tumor progression by secreting immunoglobulins, enhancing the T cell response, and directly destroying cancer cells^116,117^. pDC-secreted IFN-I and IL6 trigger the differentiation of B cells into plasma cells, in a process critical for humoral immunity^119^. Furthermore, through ligand-receptor interactions such as CD40L-CD40 and CD70-CD27, pDCs promote B cell proliferation, differentiation, and immunoglobulin production^120,121^. In addition, B cells promote INF-I secretion from pDCs^122^. However, the specific mechanism of action remains unclear. The above findings indicate that pDCs and B cells synergistically stimulate each other (**Figure 4A**), thus providing a potential basis for the development of new methods for triggering anti-tumor immune responses by priming the positive feedback of pDC–B cell crosstalk.

## pDC–NK cell crosstalk

NK cells exhibit strong cytolytic activity against tumors and can help control tumor progression^123,124^. CpG-activated pDCs produce numerous chemokines, such as CCL3, CCL4, and CCL5, in the TME, which in turn induce the migration of NK cells to tumor sites by binding the chemokine receptor CCR5 on NK cells^125^. Moreover, pDC-derived IFN-I enhances the cytolytic activity of NK cells^125^. In addition, activated pDCs in the TME stimulate NK cells *via* the OX40L–OX40 pathway and induce NK-mediated IFN-γ generation and tumor lysis, which in turn activate DCs and prime antigen-specific T cell responses. NK cells are activated by administration of TLR-stimulated pDCs to tumors^125^. However, as described above, pDCs in the TME are tolerant and may lack these functions. Tumor-infiltrating pDCs express high levels of PDL1, which engages with PD1 on NK cells and induces immunosuppression^111^. *In vitro*, anti-PDL1 antibodies resume the tumor cell lytic activity of NK cells, thereby suggesting that PDL1–PD1 ligation between pDCs and NK cells play an important role in the formation of an immunosuppressive TME^111^. A schematic representation of the crosstalk between pDCs and NK cells is shown in **Figure 4B**.

## pDC–ILC crosstalk

**Figure 5:**
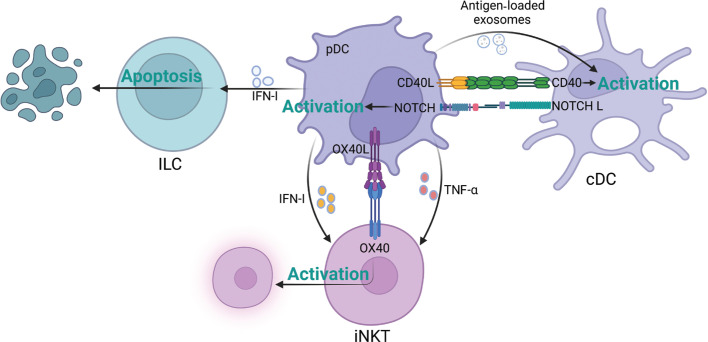
Plasmacytoid dendritic cell (pDC)–innate lymphoid cell (ILC), pDC–conventional dendritic cell (cDC), and pDC–invariant natural killer T (iNKT) cell crosstalk patterns. The figure was created with BioRender (BioRender.com).

ILCs are a newly discovered innate immune cell type with pleiotropic roles in regulating the immune response under physiological and pathological conditions^126^. ILCs comprise 3 groups: ILC1s, ILC2s, and ILC3s^126^. ILC3s secrete IL-17 and IL-22, which either promote or inhibit tumor growth, depending on the tumor type^126^. The pDCs interact with ILC3 and ILC2 (**Figure 5**). By secreting IFN-α, pDCs induce ILC3 apoptosis through the Fas cell surface death receptor-associated pathway^35,127^. In addition, pDCs inhibit the inflammatory function of ILC2, including suppression of proliferation, cytokine secretion, and ILC2 apoptosis induction, in an inflammatory disease model^128^. Thus, we speculate that the immunosuppressive role of pDCs in the TME might be partially dependent on the inhibition of the immune response of ILCs.

## pDC–DC crosstalk

Exosomes are vesicle-like structures that are secreted by cells and contain proteins, nucleic acids, lipids, and other bioactive substances with physiological or pathological functions in the body^129^. Research has demonstrated that pDCs produce exosomes under various conditions. These exosomes facilitate antigen transfer and uptake^130^. pDCs deliver antigens to cDCs *via* exosomes and subsequently cross-prime CD8^+^ T cells^130^. Although both cDC1 and cDC2 have comparable efficiency in obtaining antigens from pDCs, cDC1 plays a critical role in pDC-mediated cross-priming^130^. Moreover, IFN-I secreted by pDCs exerts potent effects on the activation and recruitment of cDCs, thereby indirectly influencing CD8^+^ T cell activation^130^. In terms of direct interaction, mDCs substantially express high levels of the Notch ligand, which binds the Notch receptor on pDCs and stimulates the immune response^131^. In turn, pDCs activate mDCs through CD40L–CD40 ligation (**Figure 5**). Thus, pDC-DC crosstalk may serve as a positive feedback loop resulting in synergistic stimulation and subsequent priming of the antitumor immune response.

## pDC–NKT cell crosstalk

NKT cells are tissue-resident, innate-like T cells that recognize lipid antigens and modulate local immune responses^132,133^. Moreover, they exhibit pronounced anticancer and anti-infection properties^134^, which are distinguished by the rapid secretion of large amounts of cytokines, including IFN-γ, IL-4, and IL-13^135^. The interaction between pDCs and invariant NKT (iNKT) cells depends on direct cell–cell contact and indirect secretory mediators (**Figure 5**). In terms of indirect interaction, by releasing TNF-α and IFN-α, CpG-activated pDCs promote survival and increase the expression of activation markers on iNKT cells^136^. In addition, the complete activation of NKT cells requires intercellular contact^137^. OX40L expressed on CpG-stimulated pDCs binds OX40 on iNKT cells, thus enhancing IFN-γ secretion by iNKT cells and IFN-I production by Pdc^135,137^. By blocking OX40L, the partial enhancement of IFN-I secretion mediated by pDCs is inhibited, thereby indicating that other costimulatory molecules are involved in the crosstalk between pDCs and NKT cells^137^. However, reports on pDC-NKT cell crosstalk have been restricted to infectious disease contexts. pDCs tend to be tolerogenic in the TME, where the interaction between pDCs and iNKT cells may differ. Thus, further studies are necessary.

## Effects of tumor biochemical factors on pDCs

**Figure 6:**
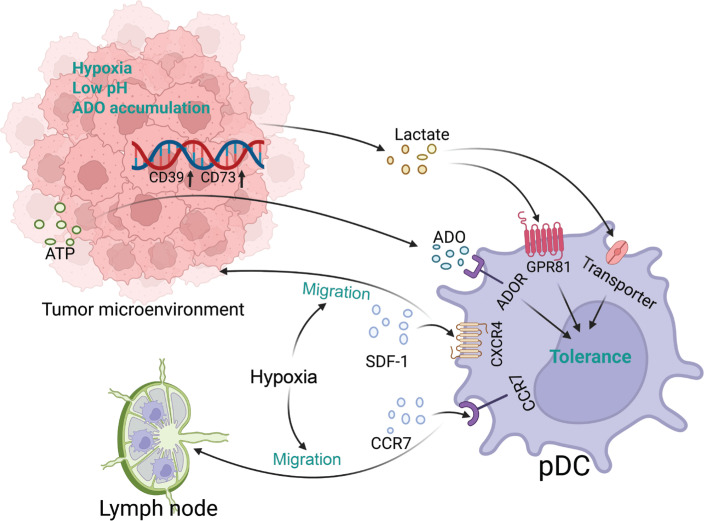
Effects of tumor biochemical factors on pDCs. The tumor microenvironment is hypoxic, acidic, and nutrient-deficient, thereby affecting pDC migration and function in multiple ways. The figure was created with BioRender (BioRender.com).

The TME has an abnormal metabolic landscape posing a substantial hurdle in ICB treatment^138,139^. Moreover, metabolic disorders in tumors lead to the development of a hypoxic, acidic environment with low glucose and amino acid levels^138,140^. This section comprehensively addresses the effects of biochemical factors, including hypoxia, lactate, and extracellular adenosine, on pDCs in the TME (**Figure 6**).

In the TME, hypoxia is a crucial regulator of pDCs, eliciting their recruitment and facilitating their immunosuppressive function^141^. Hypoxia in the TME induces pDC infiltration by upregulating the expression of several chemokines. The chemokine stroma-derived factor-1 (SDF-1) and its receptor CXCR4 play critical roles in pDC migration from peripheral blood to tumors^142^. Moreover, hypoxia promotes pDC recruitment to tumor tissues through the hypoxia-inducible factor 1α (HIF-1α)/SDF-1/CXCR4 pathway^143^. The binding of C–C chemokine ligand 19/21 (CCL19/21) to chemokine receptor 7 (CCR7) is an important enhancer of pDC homing to lymph nodes^26^. In HNSCC, hypoxia may promote pDC migration to tumor-draining lymph nodes and lymphatic metastasis by upregulating CCR7 expression^144^. CCL20 is another hypoxia-induced factor that attracts immature pDCs into tumor tissue^145^. Beyond its effects on pDC recruitment, hypoxia reprograms the differentiation and function of pDCs. As described above, E2-2 plays a crucial role in the differentiation of pDCs from progenitors and the maintenance of the pDC phenotype^14^. Weigert et al. have found that, under hypoxic conditions, activated HIF-1α promotes the expression of inhibitor of DNA binding 2 (ID2), which subsequently inhibits E2-2, and disrupts pDC maturation and differentiation^146^. In addition, the hypoxic TME upregulates HMGB1^147^, which in turn induces the tolerogenic phenotype and function of pDCs. Hypoxia also upregulates the expression levels of IDO in pDCs^148^, thus contributing to the tolerogenic status of pDCs and tumor progression. Moreover, hypoxia-induced metabolic alterations within the TME exert diverse effects on pDCs. Hypoxia reprograms the metabolism of tumor cells, thus making glycolysis the preferred modality for energy supply^149^. Glycolysis in tumor cells leads to lactate and adenosine buildup in the TME, and has profound effects on pDCs.

An important feature of tumor cell energy metabolism is the “Warburg effect,” in which glucose is fermented to produce lactate rather than carbon dioxide, even in the presence of oxygen^149^. Lactate produced by tumor cell metabolism attenuates the response of pDCs to TLR9 ligands and subsequent IFN-I secretion^150^. Lactate affects pDCs primarily *via* 2 mechanisms. In the first mechanism, lactate acts *via* the lactate receptor G protein-coupled receptor 81 (GPR81) on pDCs, thus causing intracellular calcium mobilization and subsequent inhibition of IFN-α production^150^. The second mechanism involves the direct entry of lactate into cells *via* monocarboxylate transporters expressed on pDCs^150^. Lactate entry and cytosolic accumulation in pDCs impede the CpG-induced glycolytic switch, which is essential for pDC activation after TLR stimulation^150^. In addition, lactate transported into the cytoplasm promotes tryptophan catabolism and kynurenine production by pDC. Kynurenine induces Treg expansion *via* interaction with the aryl hydrocarbon receptor, thereby leading to immunosuppression in the TME^150^.

HIF-1α is a hypoxia-regulated transcriptional activator with important functions in mammalian development, physiology, and disease pathogenesis^151^. Under hypoxic conditions in the TME, HIF-1α is translocated to the nucleus, where it transcriptionally upregulates the expression of the nucleotidases CD39 and CD73, which are critical for ATP transformation into extracellular adenosine (eADO)^152^. Tumor cell-derived eADO drives the recruitment of pDCs to tumors by interacting with the adenosine A2a receptor (A2AR) expressed on pDCs^153^. eADO also drives the immunosuppressive phenotype of pDCs, thereby leading to the accumulation of Tregs and suppression of CD8^+^ T cell proliferation and cytotoxicity, and ultimately promoting TME suppression^153^. In addition, eADO inhibits the secretion of cytokines, such as IFN-α and IL-12, from pDCs *via* A2AR, thus limiting the degree of immunogenic response^154^.

## A representative pDC crosstalk pattern in TME

**Figure 7:**
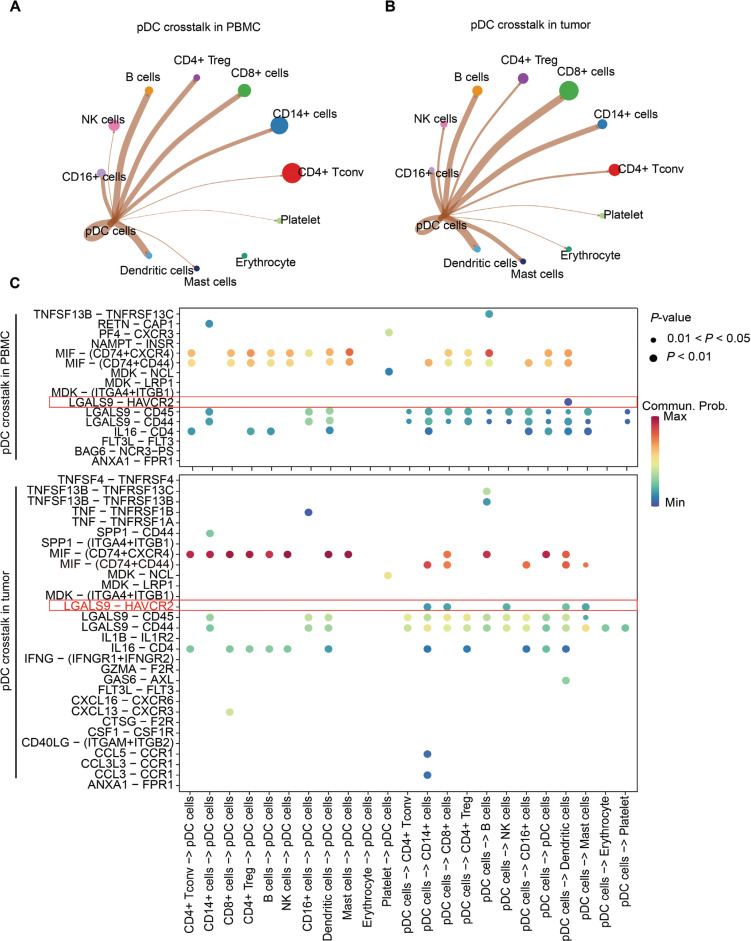
Representative pDC crosstalk with other immune cell linages in head and neck squamous cell carcinoma (HNSCC). (A, B) Circle plots showing the strength of pDC interaction with other immune peripheral blood mononuclear cells (PBMCs) and the tumor microenvironment (TME). (C) The number of ligand–receptor pairs is reflected by the line width between 2 cell types. The specific ligand–receptor pairs contributing to the crosstalk between pDCs and other cells in PBMCs (top panel) and the TME (bottom panel) are shown.

To further determine the crosstalk between pDCs and other cell lineages in the TME, we conducted CellChat analysis on publicly available single-cell RNA-Seq data from patients with HNSCC^155^. Crosstalk of pDCs with peripheral blood mononuclear cells (PBMCs) differed from that of pDCs with tumor cells. In PBMCs, pDCs interact primarily with CD16^+^ cells, B cells, CD4^+^ Tregs, CD8^+^ T cells, CD14^+^ cells, and DCs (**Figure 7A**). In the TME, pDCs interact primarily with CD16^+^ cells, NK cells, B cells, CD4^+^ Tregs, CD8^+^ T cells, CD14^+^ cells, DCs, mast cells, and other pDCs (**Figure 7B**). Moreover, the number of ligand–receptor pairs participating in pDC crosstalk in tumors is higher than that participating in pDC crosstalk with PBMCs (**Figure 7C**). In addition, the ligand–receptor pair LGALS9–HAVCR2, which is involved in pDC crosstalk, differs between PBMCs and the TME. Compared with pDCs in PBMCs, tumor-infiltrating pDCs tend to use LGALS9 to affect other cell types by binding HAVCR2. LGALS9–HAVCR2 is an immunosuppressive checkpoint pair with immunosuppressive functions^156^. Thus, LGALS9–HAVCR2 is used by tumor-infiltrating pDCs to exert immunosuppressive functions. Therefore, pDCs engage in broad crosstalk with other cell lineages, and different contexts (TME and blood) may determine the specific crosstalk modes between pDCs and other cells.

## pDC–microorganism crosstalk

Recent studies have focused on the roles of microorganisms in cancer^157,158^. As a link between innate and adaptive immunity, pDCs play important roles in sensing microorganisms and subsequently priming immune responses^14^. However, few studies have investigated the direct crosstalk between pDCs and microorganisms in cancer cells. Microorganisms exist in tumors and are important in shaping the immune microenvironment of tumors^157^. Thus, in the context of the TME, the crosstalk between pDCs and microorganisms affects tumor immunity and progression. In this section, on the basis of the existing connections between pDCs and microorganisms in infectious diseases or other models, we speculate on the possible underlying crosstalk between pDCs and microorganisms and directions for future investigation.

### pDC–virus crosstalk

Viruses account for approximately 10% of human cancers worldwide^159^. Tumor-associated viruses persist and multiply in tumor tissues^96^. pDCs play essential roles in the antiviral immune response^14^. Viral DNA and RNA are recognized by pDCs *via* TLRs, thus inducing the production of IFN-I, which subsequently primes adaptive immune responses, including the activation of mDCs, NK cells, T cells, and B cells^17^. Although, to our knowledge, no study has described direct interaction between viruses and pDCs in cancer, several reports have suggested that viruses in tumors may interact with pDCs and affect tumor immunity^101,160–162^.

HPV is associated with approximately 640,000 cancer cases and the prevalence ranks first among all virus-associated cancer^96,97^. HPV infection accounts for almost all cervical cancers and a fraction of cancers originating from the vulva, penis, and oropharynx^97^. HPV-positive cancers markedly differ from HPV-negative cancers in multiple aspects, including gene expression, mutational makeup, and the immune microenvironment^98–100^. Moreover, pDCs in the HPV-positive TME have more profound functions and immunocompetence than observed in the HPV-negative TME^101^. In addition, HPV capsid particles and E7 oncoproteins activate pDCs and induce IFN-I production by pDCs^160^. Thus, we speculate that pDCs and HPV may interact, thereby contributing to immune activation in the TME. Recently, given that HPV-related therapeutic vaccines have been actively investigated^163–165^, the crosstalk between HPV and pDCs must be studied further, and the underlying mechanism must be clarified to promote the development of new therapeutics.

Epstein-Barr virus (EBV), a gamma herpes virus, is an oncogenic virus responsible for many human cancers, including nasopharyngeal carcinoma^166^. *In vitro* and *in vivo* studies have demonstrated the central roles of pDCs in detection and protection against EBV infection^161,167^. TLR9 on pDCs recognizes EBV and triggers IFN-I production, thus inhibiting EBV entry and replication in target cells^161,162^. Kaposi’s sarcoma-associated herpesvirus (KSHV), another tumor-associated virus, also stimulates IFN-I secretion by pDCs^168^. In the TME, further investigations are necessary to determine whether the tumor-infiltrating pDCs in EBV/KSHV positive tumors might be immunocompetent and might lead to the development of immune “hot” tumors, which are candidates for tumor immunotherapy. We speculate that, in the TME, the crosstalk between pDCs and viruses might affect cancer progression. This possibility warrants further investigation and might lead to novel therapeutic strategies for cancer therapy.

### pDC–bacteria crosstalk

A previous study has highlighted the roles of bacteria in tumorigenesis, tumor immune evasion, progression, and treatment outcomes^169^. For example, gut bacteria-mediated microorganism–immune cell interactions shape the immune context within the TME and have been used to facilitate tumor immunotherapy^170–173^. In addition, intratumoral bacteria interact with and affect immune components by regulating the abundance, phenotype, and function of immune cells, including myeloid, T, B, and NK cells^174^. IFN-I secretion by pDCs can be triggered by bacteria, and can subsequently prime innate and adaptive immune responses^175,176^. In contrast, bacteria might also harm immune cells including pDCs^177–179^, thus causing immune suppression and tumor progression. In gastric cancer, the pDC population is closely correlated with specific bacteria^19^. Thus, understanding the crosstalk between pDCs and different bacteria within the TME may provide new perspectives for the development of bacteria-related immunotherapy. *Staphylococcus aureus* is a representative gram-positive commensal bacterium residing on the skin and mucosa of the human body. Studies have shown that pDCs are activated by *S. aureus* and contribute to the immune response to *S. aureus* by secreting IFN-I^175,180^. However, pDCs may also be hampered by *S. aureus* in the TME, where the eukocidin LukAB secreted by *S. aureus* targets and kills DCs^177^. Next-generation sequencing has revealed a high prevalence of *S. aureus* in the microenvironment of multiple tumor types, including breast cancer, melanoma, and pancreatic cancer^181^. Thus, given that both *S. aureus* and pDCs co-exist in the TME, the interaction between *S. aureus* and pDCs may affect the immune system and tumor development. In addition, the interaction between pDCs and other cancer-associated bacteria, including *Helicobacter pylori*, *Fusobacterium nucleatum*, *Escherichia coli*, *Bacteroides fragilis*, and *Salmonella enterica*, should be further studied to determine whether these bacteria accelerate or inhibit tumor progression.

## Conclusions and perspectives

pDCs are highly plastic and perform distinct functions in different tissues. In this review, we provided a comprehensive overview of pDC crosstalk with other components, including various cell types, biochemical factors, and microorganisms. In addition, the underlying mechanisms and functions of pDC crosstalk in the TME were comprehensively summarized. However, different tumor types and specific locations might also affect pDC function and crosstalk patterns with other cell lineages; therefore, further investigation is required. With the rapid development of single-cell RNA-Seq and spatial analysis^182^, the crosstalk between pDCs and other cell lineages has been extensively studied. In the future, we speculate that this knowledge will provide guidance for developing new strategies for targeted reprogramming of pDCs in tumors.
